# A Comparison of Standardized Letters of Evaluation for Emergency Medicine Residency Applicants

**DOI:** 10.5811/westjem.2020.12.49086

**Published:** 2020-12-21

**Authors:** David Wilson, Chaiya Laoteppitaks, Shruti Chandra

**Affiliations:** * Thomas Jefferson University, Sidney Kimmel Medical College, Philadelphia, Pennsylvania; † Thomas Jefferson University, Department of Emergency Medicine, Philadelphia, Pennsylvania

## Abstract

**Introduction:**

Medical students pursuing an emergency medicine (EM) residency are advised to obtain at least two Standardized Letters of Evaluation (SLOE). Students often complete one rotation at their home institution and at least one “away” rotation at a program separate from their home institution. The SLOE was introduced as an objective evaluation tool. The aim of this study was to determine whether there was a difference in scores between home rotation and away rotation SLOEs.

**Methods:**

We retrospectively reviewed the SLOEs of all applicants to an urban, academic EM residency program. For each SLOE, we calculated a composite score from rankings in seven “Qualifications for EM” (CS7), and converted comparative rank score (CRS) and estimated rank list position (ERP) to percentile scores. The CS7, CRS, and ERP on the home rotation SLOE were compared to those of the away SLOE using a paired t-test.

**Results:**

An evaluation of 721 applicants with at least one home SLOE and one away SLOE demonstrated a significant increase in the ERP of home rotators (P = 0.003). The data did not demonstrate a statistically significant difference in the CS7 (P = 0.69), or CRS (P = 0.97).

**Conclusion:**

Our study demonstrated that the only difference in SLOEs is that students are likely to be given a slightly higher estimated placement on the rank order list on a home SLOE. We hope this will help residency leadership with reviewing applications.

## INTRODUCTION

Medical students interested in pursuing an emergency medicine (EM) residency are advised to obtain at least two Standardized Letters of Evaluation (SLOE) from their rotations in EM.^1^,^2^ The SLOE is a letter template set forth by the Council of Emergency Medicine Residency Directors that was implemented to more effectively compare EM applicants.^3^,^4^ In order to obtain two SLOEs students often complete one rotation in EM at their home institution and one away rotation at a program other than their home institution.^1^ Most program directors (PD) cite SLOEs as an important factor in inviting applicants to interview for a residency position, demonstrating the importance of the SLOE to aspiring emergency physicians.^1^,^5^,^6^ The SLOE gives readers objective data by ranking students in tertiles in seven categories of “Qualifications for EM.” Additionally students are ranked as the top 10%, top third, middle third, or bottom third for a global assessment and estimated position on the rank list.^7^

Many aspects of the SLOE have been studied. Li et al evaluated gender biases in SLOEs and found that the narrative portions were, “relatively free of gender bias.”^8^ A study from Pelletier-Bui et al surveyed SLOE writers and found that most SLOE writers did not strictly adhere to the SLOE guidelines citing a fear that a weak evaluation could prevent an applicant from obtaining a position as an EM resident.^7^ Girzadas et al studied the precursor to the SLOE, the standardized letter of recommendation, and found that female letter writers were twice as likely to give the highest ranking to female applicants giving a basis for some ingroup bias.^9^ Although the effect of home institution on a SLOE was not studied until 2019,^10^ Program directors have long held slight preference for a SLOE from an away rotation over a SLOE from their home rotation.^6^ In 2019, Boysen-Osborn et al found that among applicants to a single, urban, academic EM residency program there was a significant difference between home and away institution SLOE scores.^10^ This confirmed for many the suspicion that students receive more favorable scores on SLOEs from their home rotation.

In this study we aimed to determine whether there was a significant difference between the objective data in SLOEs obtained from home rotations and SLOEs obtained on away rotations. We sought to determine whether any change in student performance was limited to a single category in the SLOE and to quantify the difference, if one existed.

## METHODS

We performed a single-center retrospective review of all US senior applications to the Thomas Jefferson University Hospital EM residency program through the Electronic Residency Application Service in the 2018–2019 application cycle. This study was given Thomas Jefferson University Hospital institutional board review approval before the study began.

A data abstractor collected the following for each applicant: self-identified gender; home institution; United States Medical Licensing Examination (USMLE) Step 1 score, USMLE Step 2 clinical knowledge score, whether the home rotation was first, and SLOE data. For each SLOE, we collected the location of the rotation, whether the author was an individual or committee, the scores for each question in part B “Qualifications for EM,” and the rankings in part C “Global Assessment.” The senior investigator met periodically with the abstractor to resolve any questions. Data were stored in an online secure database, OneDrive (Microsoft Corp., Redmond, WA).

We screened all SLOEs from traditional four-week EM rotations, and recorded data for all SLOEs from US senior applicants. We excluded applicants who did not have at least one home and one away rotation SLOE. In cases where a student received two SLOEs from the same rotation, only the SLOEs authored by a faculty committee, PD, or clerkship director (CD) were considered. We considered a home program to be an EM training program that was the primary affiliate of the student’s medical school or one that was available to all students from the school and did not require an application to be accepted.

The primary outcome of this study was the effect of home institution on SLOE rankings. This was done by comparing three data points for each applicant’s home and away SLOEs: a composite score of the seven “Qualifications for EM” from SLOE part B (CS7); the comparative rank score from SLOE part C1 (CRS); and estimated rank list placement from SLOE part C2 (ERP). The CS7 has a score range of 7–21 with 7 being the most favorable and 21 the least favorable. CRS and ERP are rated as top 10%, top third, middle third, or bottom third. To calculate the magnitude of any difference between home and away SLOE scores, we converted these percentiles to 10, 33, 67, and 100, respectively, rather than using ordinal numbers. The lowest percentiles are most favorable, ie, top 10% is better than top 67%. For students with more than one home or one away SLOE, a mean was calculated for each ranking, and the mean was used in the comparison. We compared scores for each outcome on the home rotation SLOE to the respective score from the away rotation SLOE using a paired t-test.

**Educational Research Capsule Summary**

What do we already know about this issue?

The away rotation Standardized Letters of Evaulation (SLOE) is often favored over the home rotation SLOE by Program Directors when reviewing applications to residency programs.

What was the research question?

This study sought to determine if there is a difference in the objective data between home and away SLOEs.

What was the major finding of the study?

The data showed that there is no difference in ratings in two major rankings between the SLOEs.

How does this improve medical education?

This study should offer residency programs more clarity when evaluating applicant SLOEs.

## RESULTS

**Figure 1:**
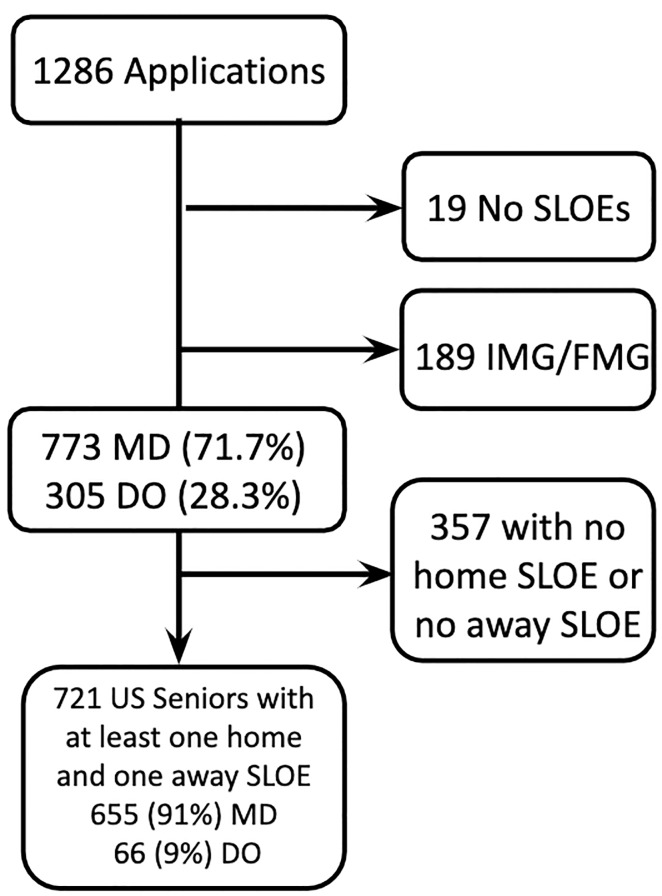
Inclusion and exclusion of study subjects. *SLOE*, Standardized Letter of Evaluation; *IMG*, international medical graduate; *FMG*, foreign medical graduate; *MD*, doctor of allopathic medicine; *DO*, doctor of osteopathic medicine.

In the 2018–2019 application cycle, there were 1823 US senior applicants to EM.^11^ The EM residency at Thomas Jefferson University Hospital received 1078 applications from US seniors. Of the received applications, 721 fit our inclusion criteria, and we recorded data for these applicants who had SLOE data from at least one home and one away rotation (Figure 1).

**Figure 2:**
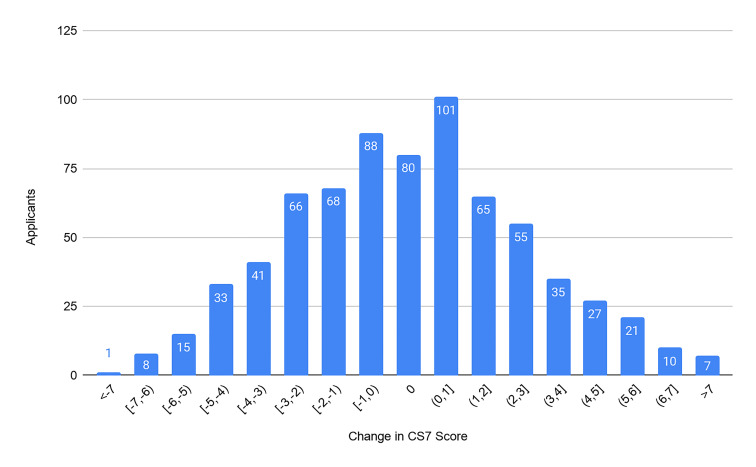
Distribution of CS7 score changes between home and away SLOEs. A negative change in score represents a more favorable score on the home rotation SLOE. *CS7*, composite score of the seven “Qualifications for Emergency Medicine.”

**Figure 3:**
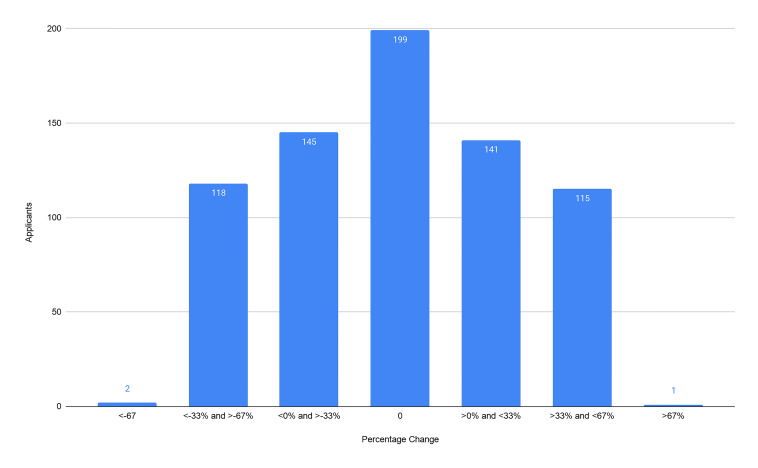
Distribution of percentile changes in comparative rank scores between home and away Standardized Letters of Evaluation (SLOE). A negative change in score represents a more favorable percentile ranking on the home rotation SLOE. *CRS*, comparative rank score.

**Figure 4:**
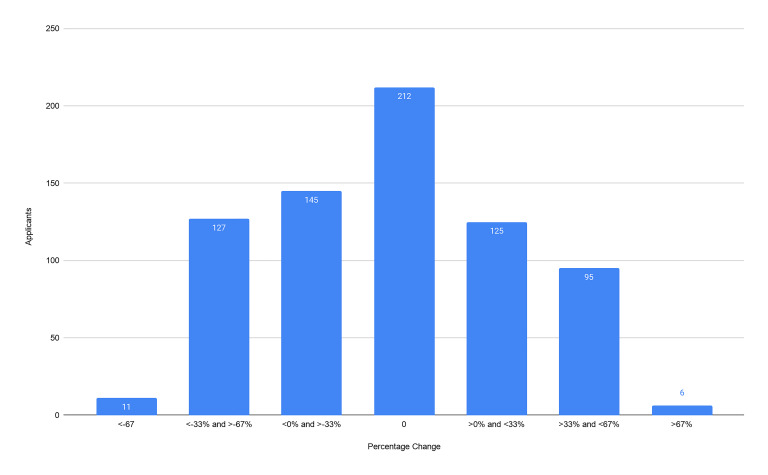
Distribution of percentile changes between estimated rank list placement between home and away rotations. A negative change in score represents a more favorable percentile ranking on the home rotation Standardized Letter of Evaluation. *ERP*, estimated rank list placement.

Our primary outcomes were the composite score of the CS7, the CRS, and the ERP. From our cohort of 721 applicants we found no significant difference between the CS7 from the home SLOE and away SLOE (*P* = 0.69). We found no significant difference between the CRS from the home SLOE and away SLOE (*P* = 0.97). We found an average of 6.9% increase in ERP (95% confidence interval, 2.4–11.5) on a home SLOE compared to an away SLOE (*P* = 0.003). For each outcome, we graphically represent the distribution of the change in scores (Figure 2–4). Further analysis looked at the same outcomes controlling for self-identified gender, degree type, and whether the home rotation was the first one completed (Table 1).

Applicants self-identifying as male gender had an average increase in ERP of 8.5% on the home SLOE, which was statistically significant (95% CI, 1.7–15.2), whereas female-identifying applicants had an average increase in ERP of 4.4% on the home SLOE, which was not statistically significant (95% CI, −0.33–9.1). With a small sample size (n = 66), osteopathic students had a small but statistically significant benefit across all three outcomes of this study. Similarly, with a small sample size (n = 83), students who completed an away rotation first had improved scores on the home rotation SLOE that were statistically significant across all three outcomes.

We conducted a secondary analysis among the cohort of applicants (n = 100) that received more than one SLOE from a single rotation including a SLOE from a committee or the institution’s standard letter writer and individual faculty. Individual faculty SLOE data differed significantly from the data from the standard letter writers and committees. The CS7 score, on average, was 1.6 points better on the SLOE from individual faculty (95% CI, 1.0,2.1). The CRS was 18.6% more favorable on the SLOE from individual faculty (95% CI, 14.2,23). The ERP was 16.5% improved from the SLOE from individual faculty (95% CI, 11.4,21.6) Each of these differences were significant with *P* < .005. Across all three outcomes, the SLOE written by the individual faculty member (as opposed to a committee letter) was statistically significantly more favorable.

## DISCUSSION

These findings are consistent with, and further build upon, the results from Boysen-Osborn et al, while reinforcing the integrity and objectivity of the SLOE. In the 2019 study, a combined score of the CRS and ERP was used as the outcome to conclude that home SLOEs were more favorable for students than away SLOEs.^10^ These findings isolate the difference in SLOEs to the ERP. Using converted percentiles rather than ordinal numbers allows for more clarity in defining the magnitude of the difference between the SLOEs. It is reassuring that there was no significant difference in CS7 or CRS as this reinforces that students are not favored by home SLOEs. However, this finding seems to be contradicted by the better ERP scores from home SLOEs. Because the difference is seen only in the ERP, it is reasonable to say that programs rank students from their institution higher than equal students from other institutions. This could be because the program already knows the applicant or has had more interaction with this student. This student may also be more likely to stay at their home institution. Additionally, given that the difference between the SLOEs is only in one rating, it is less likely that SLOEs are affected by implicit preference for home rotating students. A true preference would yield a difference across all outcomes.

The data reports a statistically significant increase in ERP from a home rotation SLOE of almost 7% (95% CI, 2.4–11). However since the SLOE stratifies by top 10%, top third, middle third, and bottom third, the 7% increase may not have placed the student in a different tier. While this could result in a different ERP score for some students, the difference may not be apparent for others. This finding shows that the objective scores on SLOEs do not vary significantly between home and away rotations. In the situation where only one home SLOE is available in the student application, especially given that the current landscape of the COVID-19 pandemic has severely limited the ability to complete away rotations, programs may regard home SLOEs as more reliable than in the past.

While the average applicant saw a modest increase in ERP from their home rotation SLOE, it appears that the bulk of this advantage fell to male-identifying students. It should be made clear that this means male-identifying students are more likely to see an increase in ERP from a home rotation SLOE, whereas female-identifying students are unlikely to see any difference in ERP between home and away SLOEs. While this study did not aim to determine whether or not a gender bias exists in the SLOEs’ objective data, this could be an area worth exploring. Despite Li et al finding that narrative portions of the SLOE are relatively free of gender bias, our findings, in addition to evidence of a gender gap in EM resident evaluations discovered by Dayal et al and a recent report showing that EM trainees are 65% male, are enough to investigate the effect of gender on the objective portions of the SLOE.^8^,^9^,^12^,^13^

Alhough potentially limited by a small sample size (n = 66), osteopathic students saw a larger benefit than allopathic students in ERP from a home rotation. Osteopathic students also had statistically significant improvement in CS7 and CRS from a home rotation. While most osteopathic institutions do not have an affiliated home EM rotation, there may be a significant advantage to osteopathic students with a home rotation SLOE. Similarly, limited by sample size (n = 83), students who completed away rotations first saw a larger improvement in home SLOE scores than the rest of the cohort. This could be explained by increased comfort in the ED and prior experience of the students who were completing a home rotation as a second or third EM rotation.

Our secondary analysis showed that SLOEs written by individual faculty members who were not standard SLOE writers varied significantly from SLOEs written by standard SLOE writers for the same applicant during the same rotation. Committee SLOEs are already recognized as superior to individual SLOEs. Individual SLOEs are treated as classic narrative letters of recommendation; our data simply supports this.

The SLOE has evolved over the years and remains an integral part of the EM residency application. Many PDs continue to use USMLE Step 1 scores as part of the residency selection process because of its utility for predicting future board certification.^1^,^6^,^14^ Given that Step 1 score-reporting will soon become pass/fail, it would be helpful to correlate SLOEs with future board certification, and perhaps it is time for the SLOE to undergo a new evolution to become predictive in such a manner.

## LIMITATIONS

Our study had several limitations. Although our sample did account for almost 60% of all US seniors applying into EM, we only reviewed the SLOEs of applicants to a single institution, which could have skewed the data. We used a definition for a home rotation that may not be uniform among all residency programs reviewing applications. While some institutions have obvious relationships with EM residency programs, some are more covert. Our study did not take into account whether the letter was written by an individual or SLOE committee, and did not consider the geographic location of away rotations.

## CONCLUSION

This study explores the difference between home rotation and away rotation SLOEs. In this study we concluded that the only difference in SLOEs was that students were likely to be given a slightly higher estimated placement on the rank order list on a home SLOE. Further topics of study could consider geographic location of the away rotations and their proximity or relationship to the home institution or consider the first SLOE vs the second SLOE in addition to home vs away letters.

## Figures and Tables

**Table 1:** Primary outcomes comparing home and away Standardized Letters of Evaluation. A negative difference translates to a more favorable score from the home rotation. Outcomes also stratified by degree type, self-identified gender, and whether the home rotation was the first rotation completed.

	n (%)	Average USMLE Step 1 Score	Average USMLE Step 2 CK Score	Change in CS7 (95% CI)	Change in CRS (95% CI)	Change in ERP (95% CI)
Total	721	229	245	0.04 (−0.18,0.27)	−0.04% (−2.1,2.0)	−6.9%* (−11.5,−2.4)
MD	655 (90.8)	229	245	0.16 (−0.07,0.39)	1.0% (−1.2,3.2)	−5.8%* (−10.6,−1.1)
DO	66 (9.2)	228	240	−1.1* (−1.9,−0.3)	−10.3%* (−16.5,−4.1)	−17.7%* (−33.6,−1.78)
Male	449 (63.1)	231	244	0.13 (−0.15,0.43)	0.23% (−2.4,2.9)	−8.5%* (−15.2,−1.7)
Female	272 (37.7)	227	246	−0.11 (−0.47,0.25)	−0.48% (−3.8,2.8)	−4.4% (−9.1,0.33)
Home First	638 (88.5)	229	245	0.17 (−0.08,0.41)	1.1% (−1.14,3.32)	−5.4%* (−10.1,−0.62)
Away First	83 (11.5)	227	242	−0.88* (−1.45,−0.30)	−8.7%* (−13.8,−3.7)	−19.0%* (−34.3,−3.63)

* *P*<.05

*USMLE*, United States Medical Licensing Examination; *CK*, clinical knowledge, *CI*, confidence interval; *CS7*, composite score of the seven “Qualifications for EM”; *CRS*, comparative rank score; *ERP*, estimated rank list placement; *MD*, doctor of allopathic medicine; *DO*, doctor of osteopathic medicine.
